# Association of Vital Signs and Process Outcomes in Emergency Department Patients

**DOI:** 10.5811/westjem.2019.1.41498

**Published:** 2019-04-16

**Authors:** Nicole R. Hodgson, Karl A. Poterack, Lanyu Mi, Stephen J. Traub

**Affiliations:** * Mayo Clinic Hospital, Department of Emergency Medicine, Phoenix, Arizona; † Mayo Clinic Hospital, Department of Anesthesiology, Phoenix, Arizona

## Abstract

**Introduction:**

We sought to determine the association of abnormal vital signs with emergency department (ED) process outcomes in both discharged and admitted patients.

**Methods:**

We performed a retrospective review of five years of operational data at a single site. We identified all visits for patients 18 and older who were discharged home without ancillary services, and separately identified all visits for patients admitted to a floor (ward) bed. We assessed two process outcomes for discharged visits (returns to the ED within 72 hours and returns to the ED within 72 hours resulting in admission) and two process outcomes for admitted patients (transfer to a higher level of care [intermediate care or intensive care] within either six hours or 24 hours of arrival to floor). Last-recorded ED vital signs were obtained for all patients. We report rates of abnormal vital signs in each group, as well as the relative risk of meeting a process outcome for each individual vital sign abnormality.

**Results:**

Patients with tachycardia, tachypnea, or fever more commonly experienced all measured process outcomes compared to patients without these abnormal vitals; admitted hypotensive patients more frequently required transfer to a higher level of care within 24 hours.

**Conclusion:**

In a single facility, patients with abnormal last-recorded ED vital signs experienced more undesirable process outcomes than patients with normal vitals. Vital sign abnormalities may serve as a useful signal in outcome forecasting.

## INTRODUCTION

“Vital signs are vital” is a common refrain in emergency medicine. Emergency physicians (EP) are taught early in their careers that persistent tachycardia at discharge should give them pause and that a hypotensive patient often isn’t suitable for admission to a floor bed.

Previous studies support this traditional teaching. In discharged elderly patients, specific vital sign abnormalities (systolic blood pressure [SBP] < 97 millimeters of mercury [mmHg], heart rate > 101 beats per minute, body temperature > 37.3° C, and pulse oximetry < 92 SpO_2_) were associated with twice the odds of admission within seven days of emergency department (ED) discharge, with the greatest odds found in patients with two or more abnormal vital signs.^1^ Other studies focused on admitted patients, with mixed findings. Some found only tachypnea on admission to be correlated with upgrade in level of care within 24 hours,^2^ whereas others found tachypnea, tachycardia, and hypoxia both on arrival to and departure from the ED, along with hypotension or hypertension on departure from the ED, to be associated with activation of a dedicated rapid-response team within 12 hours (a surrogate marker for patient decompensation).^3^

If vital sign abnormalities are consistently associated with undesirable process outcomes, artificial intelligence (AI) programs could notify EPs prior to final disposition. We sought to determine the association of abnormal vital signs with ED process outcomes in a large population of both discharged and admitted patients in a single hospital.

## METHODS

This was a retrospective analysis of routinely gathered ED operational data. This project was part of a quality improvement effort, and our institutional review board process identified it as exempt, with a waiver of the requirement for informed consent.

The Mayo Clinic Arizona ED is a tertiary care facility in Phoenix, Arizona. During the study period, there were 24 rooms and up to nine hallway spaces. There is no emergency medicine training program, but resident physicians from multiple services rotate through the ED and assist in the evaluation of approximately 5% of patients. The ED was staffed 24 hours per day with residency-trained EPs. There was no fast track and no mechanism for ED observation. Patients were allocated to physicians via rotational assignment, which removes essentially all physician discretion as to which patients a provider will evaluate.^4^

We analyzed all recorded eligible patient visits between July 1, 2012, and June 30, 2017. We defined eligible visits as those involving patients who were 18 years of age or older who were either discharged home without need for ancillary services (Group 1) or admitted to a floor (ward) bed (Group 2). We excluded patients discharged with ancillary services and admissions to intermediate or intensive care unit (ICU) beds.

Nursing staff collected vital signs from monitors, validating them for upload into the electronic health record (EHR) (Cerner Millennium; Cerner®, Kansas City, Missouri). For every visit, we obtained the last-recorded ED value for pulse, BP, respiratory rate, and temperature. These values were not necessarily obtained simultaneously. We excluded visits missing one or more vital signs. We also excluded visits with vital signs unlikely to be accurate entries (heart rate < 30 or > 200, respiratory rate (RR) < 5 or > 60, temperature < 30 or > 45, SBP < 50 or > 300, mean arterial pressure (MAP) < 20 or > 200). These values were chosen based upon an initial review of outliers in an attempt to exclude clinically improbable scenarios.

We defined tachycardia as a pulse ≥ 100. We defined hypotension as a SBP < 90 mmHg or a MAP of < 65 mmHg. We defined tachypnea as a RR > 20 breaths/minute. We defined fever as a temperature ≥ 38 °C.

For Group 1, the outcomes of interest were returns within 72 hours of discharge from the ED and returns within 72 hours of discharge from ED that were subsequently admitted to our hospital or transferred to another hospital with the intention of admission. For Group 2, the outcomes of interest were transfer to a higher level of care (intermediate unit or ICU) within six hours or 24 hours after arrival to a floor bed.

We examined the frequency of vital sign abnormalities in each group and used Pearson’s chi-square to test the association between vital signs and outcomes. We considered p values < 0.05 to be statistically significant. We report the relative risk of meeting a process outcome for each individual vital sign abnormality. Treating each vital abnormality as a diagnostic test to examine the precision with which it can identify a process measure, we calculated positive and negative predictive values. Data were abstracted by one investigator (SJT) from a custom operations report in Microsoft Excel (Redmond, Washington) format. We used SAS Studio 3.7 (SAS Institute, Inc, Cary, North Carolina) for the analysis.

## RESULTS

We show our study flowchart in the figure, and report results in Table 1a–d. Patients with tachycardia, tachypnea, or fever more commonly met criteria for every process outcome compared to patients without these abnormalities. Patients who were hypotensive at admission more frequently required transfer to a higher level of care within 24 hours.

## DISCUSSION

Indirect ICU admissions (patients initially admitted to a floor or ward bed and later upgraded to ICU) are associated with negative patient outcomes, including increased mortality at 72 hours^4^ and at 30^4^,^5^ and 60^6^ days. ED returns with admissions may have increased mortality and morbidity as well; one study found a 7.1% mortality and 21.7% complication rate in patients with 72-hour revisits resulting in admission.^7^

Recent work has focused on the development of predictive tools based on ED vital signs to assist EPs in identifying patients at risk for decompensation.^8^,^9^ One example, the PeRRT (Predicting Early Rapid Response Team) score, incorporates vital signs (among other data) to predict which patients would trigger a rapid response activation during the first 12 hours of admission.^10^ Despite the associations of vital signs with negative process outcomes, most patients discharged or admitted to the floor with abnormal vital signs did not have negative outcomes, limiting the utility of vital signs alone as a predictive tool. This suggests a need to incorporate additional factors in any predictive algorithm. Age, serum bicarbonate, and lactic acid have separately been shown to be associated with inpatient deterioration.^11^

The future application of AI to ED patient data could improve predictive models to a point where they become more accurate. ED triage-based AI programs have shown promise. One algorithm incorporating age, sex, arrival mode, chief complaint, active problems and arrival vital signs showed equivalent or improved ability to detect patients needing ICU admissions, emergent procedures, and hospital admission when compared to the Emergency Severity Index.^12^ Similarly, AI may soon be able to prospectively identify patients at risk of both inpatient and outpatient deteriorations. Although our vital sign data by itself was insufficient to create a sensitive and specific algorithm, the addition of other clinical data could lead to the development of a useful AI tool to alert EPs to potentially unsafe dispositions.

## LIMITATIONS

We did not analyze pulse oximetry data. It is difficult to extract data from our EHR regarding supplemental oxygen status, making oxygenation values difficult to interpret. Additionally, we did not examine hypertension or hypothermia. We limited our analysis to those who were discharged home with no ancillary services and those who were admitted to a floor bed. Although our methodology prevents extrapolation to other groups (such as patients discharged to home hospice or patients admitted to an intermediate setting), it removed several potentially confounding factors.

We did not account for “scheduled” ED visits, such as encounters for suture removal or wound re-checks. We believe this had little impact on our data, however, as most patients in our healthcare system present to their primary physicians for this follow-up. Additionally, we were unable to account for discharged patients who may have presented to other EDs within 72 hours, as this information was not readily obtainable in our EHR.

## CONCLUSION

Patients with tachycardia, tachypnea or fever recorded as their final ED vital sign more frequently experienced undesirable process outcomes. Despite these associations, most admitted patients with abnormal vital signs did not require upgrades in level of care, and most discharged patients with abnormal vital signs did not have a return visit or a return visit with admission. Vital sign abnormalities may serve as a useful signal in outcome forecasting; however, a more nuanced model that combines vitals data with other factors is needed to make a clinically useful predictive model. AI may soon provide the necessary technology to create this tool.

## Figures and Tables

**Figure:**
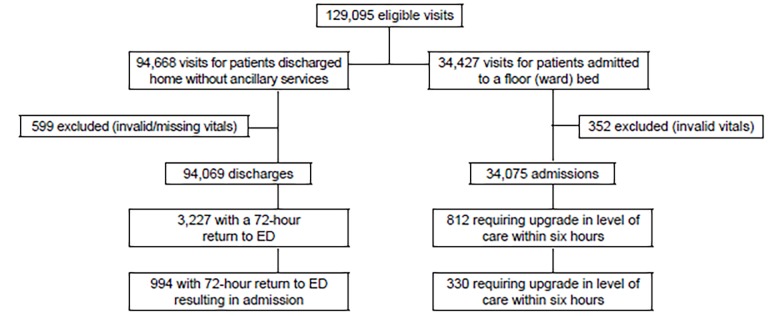
Study flowchart. *ED*, emergency department.

**Table 1a:** Vital sign abnormalities and 72-hour returns in discharged patients.

Vital sign	Return (n=3227)	No return (n=90842)	P-value	RR (95% CI)	PPV	NPV
Hypotension	25	557	0.2501	1.25 (0.85–1.84)	4.30%	96.57%
Tachycardia	198	4662	0.0114*	1.20 (1.04–1.38)	4.07%	96.60%
Tachypnea	184	4371	0.0206*	1.19 (1.03–1.37)	4.04%	96.60%
Fever	42	576	<0.0001*	1.99 (1.49–2.68)	6.80%	96.59%

*RR*, relative risk; *CI*, confidence interval; *PPV*, positive predictive value; *NPV*, negative predictive value.

* Denotes statistically-significant P-values.

**Table 1b:** Vital sign abnormalities and 72-hour return with admission in discharged patients.

Vital sign	Return+admit (n=994)	No return+admit (n=93075)	P-value	RR (95% CI)	PPV	NPV
Hypotension	11	571	0.0486*	1.80 (1.00–3.24)	1.89%	98.95%
Tachycardia	73	4787	0.0018*	1.45 (1.15–1.84)	1.50%	98.97%
Tachypnea	89	4466	<0.0001*	1.93 (1.56–2.40)	1.95%	98.99%
Fever	25	593	<0.0001*	3.90 (2.64–5.76)	4.05%	98.96%

*RR*, relative risk; *CI*, confidence interval; *PPV*, positive predictive value; *NPV*, negative predictive value.

* Denotes statistically-significant P-values.

**Table 1c:** Vital sign abnormalities and six-hour upgrades in admitted patients.

Vital sign	Six-hour upgrade (n=330)	No six-hour upgrade (n=33745)	P-value	RR (95% CI)	PPV	NPV
Hypotension	10	593	0.0809	1.73 (0.93–3.24)	1.66%	99.04%
Tachycardia	87	3992	<0.0001*	2.63 (2.07–3.36)	2.13%	99.19%
Tachypnea	96	5444	<0.0001*	2.11 (1.67–2.68)	1.73%	99.18%
Fever	29	1100	<0.0001*	2.81 (1.93–4.10)	2.57%	99.09%

*RR*, relative risk; *CI*, confidence interval; *PPV*, positive predictive value; *NPV*, negative predictive value.

* Denotes statistically-significant P-values.

**Table 1d:** Vital sign abnormalities and 24-hour upgrades in admitted patients.

Vital Sign	24-Hour upgrade (n=812)	No 24-hour upgrade (n=33263)	P-value	RR (95% CI)	PPV	NPV
Hypotension	26	577	0.0017*	1.84 (1.25–2.69)	4.31%	97.65%
Tachycardia	188	3891	<0.0001*	2.22 (1.89–2.60)	4.61%	97.92%
Tachypnea	214	5326	<0.0001*	1.84 (1.58–2.15)	3.86%	97.90%
Fever	55	1074	<0.0001*	2.12 (1.62–2.77)	4.87%	97.70%

*RR*, relative risk; *CI*, confidence interval; *PPV*, positive predictive value; *NPV*, negative predictive value.

* Denotes statistically-significant P-values.
